# New Insights into Dietary Pterostilbene: Sources, Metabolism, and Health Promotion Effects

**DOI:** 10.3390/molecules27196316

**Published:** 2022-09-25

**Authors:** Sanjushree Nagarajan, Sundhar Mohandas, Kumar Ganesan, Baojun Xu, Kunka Mohanram Ramkumar

**Affiliations:** 1 Department of Biotechnology, School of Bioengineering, SRM Institute of Science & Technology, Kattankulathur 603 203, India; 2 School of Chinese Medicine, LKS Faculty of Medicine, University of Hong Kong, 10 Sassoon Road, Hong Kong 999077, China; 3 Food Science and Technology Programme, Department of Life Sciences, BNU-HKBU United International College, Zhuhai 519087, China

**Keywords:** pterostilbene, resveratrol, antioxidant, bioavailability, cancer, diabetes, Nrf2

## Abstract

Pterostilbene (PTS), a compound most abundantly found in blueberries, is a natural analog of resveratrol. Several plant species, such as peanuts and grapes, produce PTS. While resveratrol has been extensively studied for its antioxidant properties, recent evidence also points out the diverse therapeutic potential of PTS. Several studies have identified the robust pharmacodynamic features of PTS, including better intestinal absorption and elevated hepatic stability than resveratrol. Indeed, due to its higher bioavailability paired with reduced toxicity compared to other stilbenes, PTS has become an attractive drug candidate for the treatment of several disease conditions, including diabetes, cancer, cardiovascular disease, neurodegenerative disorders, and aging. This review article provides an extensive summary of the nutraceutical potential of PTS in various disease conditions while discussing the crucial mechanistic pathways implicated. In particular, we share insights from our studies about the Nrf2-mediated effect of PTS in diabetes and associated complications. Moreover, we elucidate the important sources of PTS and discuss in detail its pharmacokinetics and the range of formulations and routes of administration used across experimental studies and human clinical trials. Furthermore, this review also summarizes the strategies successfully used to improve dietary availability and the bio-accessibility of PTS.

## 1. Introduction

Pterostilbene (PTS) (trans-3,5-dimethoxy-4′-hydroxystilbene) is a natural polyphenol and a dimethyl ether analog of resveratrol [1]. PTS is produced by plants as a secondary metabolite that serves to respond to environmental challenges, including UV radiation, drought, fluctuating temperature extremes, grazing pressures, and fungal infections, and PTS serves as an important mediator of disease resistance [2,3]. Similar to resveratrol, PTS also behaves as a phytoalexin, conferring crucial anti-pathogenic defense to plants [4,5]. The daily consumption of PTS is determined by its dietary intake. Based on the type of blueberry ingested, the content of PTS is estimated to range from 99 ng to 520 ng/gram of fruit [6]. Though berries are the most evident source of PTS, it has been reported to be present in various other food sources, including peanuts.

As a natural dietary component, PTS has been documented to exhibit an increased bioavailability compared to other stilbene compounds, which further highlights the need to study the clinical potential of this compound in medical conditions [7,8]. Various evidence has demonstrated the effect of PTS in countering oxidative damage and inflammation, imparting preventive and therapeutic benefits in experimental disease models [4,7,9]. Indeed, through its antioxidant and anti-inflammatory activity, PTS has been reported to regulate pathogenic pathways associated with carcinogenesis, hematologic diseases, neurological disorders, vascular dysfunction, aging disorders, and diabetes [7]. 

Among the identified polyphenols, resveratrol has been determined to exhibit relatively poor oral bioavailability and undergoes rapid first-pass metabolism. On the contrary, methylated polyphenols such as PTS have been documented to possess better intestinal absorption and elevated hepatic stability. Considering the potential drawbacks that are exhibited due to the unfavorable pharmacodynamics of resveratrol, much focus has shifted towards understanding and characterizing the pharmacokinetics and therapeutic potential of PTS. Overall, this review provides evidence that PTS is a promising, novel, potent, and safe drug candidate for treating various diseases and disorders.

## 2. Potential Dietary Sources of PTS

Various human diet crops have been documented to produce PTS at varying levels (Table 1). Red sandalwood, also referred to as Heartwood (*Pterocarpus santalinus*), was the first identified source of PTS [3]. Of note, contrary to flavonoids, which are produced by many plants, stilbenes are synthesized by only a few plant species (Table 1). PTS has been reported to be abundantly found in Indian Kino (*Pterocarpus marsupium*), *Guibourtia tessmannii*, and *Vaccinium* spp. berries and at relatively lower levels in the leaves of grape (*Vitis vinifera*) and blueberry fruits [9,10]. The concentration ranges from 9.9 to 15.1 mg/kg of blueberries, 0.2 to 4.7 mg/g of the weight of the skin of fungus-infected grapes, 99 to 151 ng/g dried sample of rabbit-eye blueberry (*Vaccinium ashei*), and 520 ng/g dried sample of deerberries (*Vaccinium stamineum*) [11]. Additionally, peanut (*Arachis hypogaea*) has also been identified as a source of PTS [12] (Table 1). However, it should be noted that the amount of PTS in many of these food sources may be insufficient to provide documented health benefits. Dietary supplements of the formulated pure compound offer a solution to provide sufficient nutritional levels. Moreover, considering the growing interest in PTS as a promising nutraceutical compound, further research that aims to identify the compound in fresh and processed food products by employing standardized extraction and analytical methods is warranted.

### Biosynthesis and Nutraceutical Availability

The biosynthesis of PTS is facilitated through the conversion of the amino acids phenylalanine or tyrosine, which are products of the shikimate pathway. These amino acids are converted to coumarate and then to p-coumaroyl-CoA, resulting in the production of precursor stilbenes. While stilbene synthase converts precursor stilbenes to resveratrol, an O-methyl transferase further methylates two of the resveratrol hydroxyl groups of resveratrol to form PTS [15].

The availability of PTS in plants was found to vary within species based on various genetic and environmental factors [16]. Interestingly, fungal infection induced the elevated production of PTS in certain food crops, such as grapes [17]. Further, exposure to ultraviolet light amplified the production of resveratrol, while it reduced the production of PTS in grapevines [18]. The concentration of PTS was observed to be relatively high in the fruit skins or epidermal tissues of plants. This could be an evolutionarily conserved effect to protect the plant from harmful microbes that could cause infection through the penetration of the plant epidermis [19]. The variability in the nutraceutical availability within a single plant indicates that different conditions are required to produce resveratrol and PTS. The transgenic alteration of phenolic metabolism has been proposed as a key strategy to improve the yield of PTS from dietary sources. Notably, PTS was produced even by species that do not produce the compound through transgenic alteration. Tobacco (*Nicotiana tabacum* L.) and *Arabidopsis thaliana* (L.) Heynh. were transformed to produce PTS by employing a stilbene synthase transgene from peanut along with an O-methyltransferase transgene from *Sorghum* bicolor (L.) Moench [10]. Of note, the production of PTS in tobacco was accompanied by reduced flavonoid levels, indicating that stilbenes and flavonoids compete for p-coumaroyl-CoA in their biosynthesis pathways. 

Additionally, PTS production has been reported to be amplified in food crops that already produce the compound through metabolic engineering. In grapevine cell cultures that were transformed to constitutively express *V. vinifera* O-methyltransferase, PTS production was observed to be elevated [20]. An effort was also made to employ stilbene-synthesizing gut bacteria to provide a constant PTS supply to animals without the dietary ingestion of the compound [21]. 

## 3. Analytical Aspects

Stilbenoids are a class of non-flavonoid polyphenolic compounds with a molecular weight of approximately ∼200–300 g/mol [22]. Some of the members of the stilbene family include resveratrol, PTS, and 3′-hydroxy PTS [22]. Stilbenoids are characterized by a C6-C2-C6 skeleton and the presence of phenyl groups that are linked by ethene double bonds [22]. With a molecular weight of 256.29 g/mol, PTS is a 3,5-dimethoxy analog of resveratrol [22]. PTS is a methoxybenzene and a diether due to the presence of trans-stilbene with methoxy groups at the 3′ and 5′ positions and a hydroxy group at the 4′ position [22]. Even though PTS exists in both cis and trans structures, it is most abundant in its monomeric, lipid-soluble trans form [9]. Upon the equimolar administration of resveratrol and PTS in rats, systemic exposure and the plasma concentration (higher C_max_ and AUC_0–inf_) were greater in PTS when compared to resveratrol, whereas the total body clearance of resveratrol was greater than that of PTS [4]. The two methoxy groups in PTS have been identified as responsible for increasing its oral absorption and bioavailability compared to resveratrol [22].

## 4. Pharmacokinetics

PTS is often consumed in berries, grapes, nuts, and wine. In the form of a dietary polyphenol, PTS has exhibited safety at a high dose of 3 g/kg body weight for 28 days, not leading to any toxicity in mice [23]. Indeed, PTS was found to exhibit dose-dependent pharmacokinetics. An increased intravenous dose (25 mg/kg) reduced the elimination of PTS and was associated with almost twice the rate of reduced clearance due to the saturation of PTS metabolism [24]. When administrating PTS through the oral route, escalating the dosage from 15 mg/kg to 30 or 60 mg/kg doubled the bioavailability (F) and prolonged the mean residence time. This trend is attributed to the absorption and limited elimination of the compound [25]. At a dose of 2.5 mg/kg, rapid absorption and moderate bioavailability were observed with the sublingual administration of PTS [25].

### 4.1. Absorption

PTS has high membrane permeability due to its specific features, including lipophilicity, low polar surface area, rotatable bonds, and hydrogen-bond acceptors and donors [3]. PTS has poor solubility in water (around 21 µg/mL), which can be overcome by solubilizing it with piperazine at a 2:1 stoichiometric molar ratio. Piperazine–PTS cocrystals had six times greater solubility than PTS alone [26]. Moreover, administering PTS after a meal was observed to increase its oral absorption, as food consumption leads to the acceleration of bile secretion, which enhances the aqueous solubility of drugs co-administered with food [24]. The bioavailability of PTS has also been reported to be enhanced by using it in combination with a 2-hydroxypropyl-β-cyclodextrin (15 mg/kg) solution [25]. Notably, following oral administration, PTS exhibited higher bioavailability than resveratrol, with greater total plasma levels of its metabolites and parent compound [4].

### 4.2. Distribution

The apparent volume of distribution (measured by the V_ss_ value) of PTS (V_ss_ = 5.3 L/kg) following intravenous dosing in rats was greater than that of the total body water (V_ss_ = 0.7 L/kg), indicating substantial tissue distribution [4]. The distribution of PTS has been found in the liver, kidney, heart, lungs, and brain [27]. The primary metabolites of PTS are the sulfate and glucuronide conjugates [4]. After intravenous administration in male rats, sulfate conjugates seem to be more extensive than glucuronide conjugates [4]. After 1–2 h of oral administration, the entero-hepatic recycling of the metabolite with an increased concentration of glucuronidated PTS was reported [2,28].

### 4.3. Metabolism

The cytochrome P450 superfamily comprises phase I enzymes, which are in charge of the biotransformation of compounds to reduce their toxicity and increase their polarity, which in turn facilitates the elimination of the drug from the system. Meanwhile, phase-II-mediated enzymes are involved in the biotransformation of xenobiotic metabolites that are the products of phase I metabolism. Notably, phase II detoxification enzymes are crucially involved in promoting drug conjugation and antioxidant reactions [3]. PTS is predominantly cleared through the phase-II-drug-metabolizing pathway by glucuronidation and sulfation [3]. The involvement of this metabolizing pathway is supported by increased concentrations of glucuronide and sulfate PTS conjugates in the systemic circulation when compared to its parent compound form [4].

### 4.4. Excretion

Around 99% of PTS is excreted through non-renal pathways, while 0.219% goes through the hepatic pathway [8]. A very small fraction of it was found to be excreted in urine [8]. When comparing intravenous doses of resveratrol and PTS in male rats, the clearance of PTS was much less than that of resveratrol, indicating a longer therapeutic availability [3]. Interestingly, the analysis of the urine samples in cannulated rats identified the parent PTS and its glucuronidated metabolite, which had been previously identified only in the systemic circulation [8]. Increasing the dose of PTS from 2.5 mg/kg to 25 mg/kg decreased the clearance rate by almost half, which is attributed to the saturation or partial saturation of PTS metabolism [24]. 

### 4.5. Toxicity

The administration of PTS, even in high doses, was observed to be nontoxic in mouse models. Four groups of mice were fed PTS doses ranging from 0 to 3000 mg/kg body weight/day for 4 weeks, and there were no significant alterations in the consumption of food or water or in weight gain [23]. Additionally, the in vivo administration of PTS attenuated tumorigenesis and metastasis with negligible toxicity [29]. Pharmacologically, PTS, when intravenously administered, has been noted to be safe, as the compound did not exhibit toxicity specific to any organ [30]. In humans, PTS has been observed to exhibit safety at doses up to 250 mg/day [31].

## 5. Major Pathways Associated with PTS

### 5.1. Antioxidative Pathway: Activation of Nrf2 Signaling

**Figure 1:**
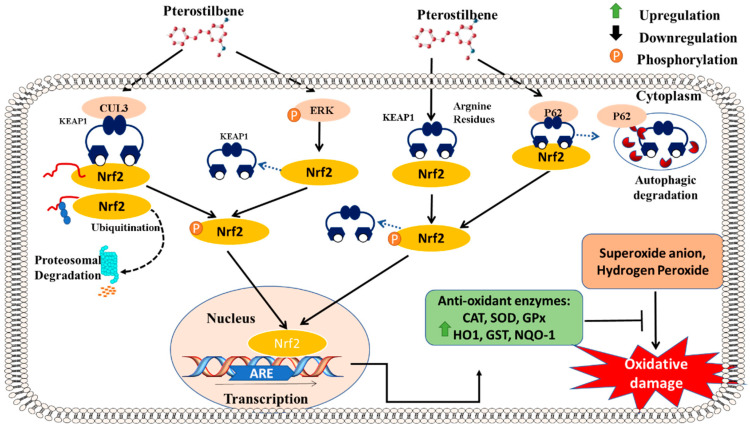
**Nrf-2-mediated antioxidant pathway of pterostilbene:** Activation and phosphorylation of Nrf-2 signaling is the major mechanism through which the antioxidative response is induced by PTS. Ubiquitination mediated by Cullin-3 (CUL-3) leads to the proteasomal degradation of Nrf-2. PTS inhibits the ubiquitin–proteasome system, thereby increasing the accumulation of Nrf-2. PTS also enables the phosphorylation of Nrf-2, which is critical in the nuclear translocation of the transcription factor. Moreover, PTS phosphorylates and activates the ERK signaling pathway, which mediates the dissociation of Keap-1, resulting in Nrf-2 activation. Furthermore, PTS stimulates the binding of Keap-1 and p62, which enhances the activation of Nrf2. Following its activation and nuclear translocation, Nrf-2 binds to ARE and induces the expression of antioxidant enzymes, which in turn critically attenuate oxidative damage in host cells.

The antioxidant activity of PTS has been extensively studied and implicated in anti-carcinogenesis, the modulation of neurological disorders, the attenuation of vascular diseases, and diabetes management [7]. Extensive evidence has indicated that PTS reduces oxidative stress by attenuating the production of the superoxide anion and hydrogen peroxide, which are implicated in the initiation and progression of various pathogenic processes [7]. Nrf2, a nuclear transcription factor, is one of the major players in regulating cytoprotective and antioxidant genes, which also includes phase II metabolic and antioxidant enzymes [32]. The stimulation of Nrf2 signaling has been identified to produce anti-cancer, anti-diabetic, cardioprotective, and neuroprotective effects [33]. Kelch-like ECH-associated protein 1 (Keap-1) is a negative regulator of Nrf2 and targets the transcription factor for ubiquitylation and degradation. Our lab investigated the protective properties of PTS in pancreatic β-cell apoptosis through an Nrf2-mediated mechanism [32] (Figure 1). We found that PTS activates the Nrf2 pathway, thereby triggering the expression of Nrf2 downstream target genes to facilitate cellular protection in INS-1E cells. In particular, we demonstrated that PTS binds to the arginine residues of Keap-1 and facilitates its disassociation from Nrf2 [34]. Interestingly, PTS has also been reported to mediate the recruitment and interaction of the P62 autophagic cargo with Keap-1, thereby inducing the autophagic degradation of Keap-1 [35]. The major downstream targets activated following PTS administration were antioxidative enzymes, including HO 1, SOD, catalase, and GPX. Moreover, we observed the upregulation of anti-apoptotic gene expression, with the associated downregulation of the expression of the pro-apoptotic mediators Bax (Bcl-2 associated X protein) and caspase-3. Our evidence was strongly suggestive of the protective effect of PTS administration against hyperglycemia-induced oxidative damage in pancreatic β-cells [32] (Figure 1). 

Our lab inferred that PTS protects β-cells in Streptozotocin (STZ)-induced diabetic mice, an effect accompanied by the induction of Nrf2 and the consequent upregulation of its target genes. We also investigated the anti-peroxidative role of PTS in the STZ-induced diabetic model. In addition, PTS normalized the circulatory concentration of VLDL and LDL while reducing lipid peroxidation in STZ-induced diabetic mice. Notably, the livers of diabetic mice indicated collapsed hepatic microvesicles on H&E staining due to altered lipid metabolism [36]. PTS administration reduced structural and functional alterations in the hepatic tissue, indicating its protective function in diabetic dyslipidemia mediated via Nrf2 activation [37] (Figure 1). 

By countering oxidative damage, PTS treatment inhibited human retinal endothelial cell proliferation and delayed the progression of diabetic retinopathy [38]. In the livers of IUGR piglets, PTS attenuated liver injury caused by Nrf2 activation and the consequent induction of the antioxidant response [39]. Notably, adrenocorticotropic hormone (ACTH) was observed to interfere with Nrf2 signaling in metastatic cells. PTS reduced ACTH activity and was effective against various melanoma cell lines, including MelJuso, A2058, and MeWo [30]. PTS blocked cellular inflammation and oxidative stress in azoxymethane-induced colon carcinogenesis, thereby reducing tumorigenesis. Through the activation of Nrf2, PTS countered the induction of NF-κB (nuclear factor-kappaB) and diminished the levels of oxidative stress mediators, including inducible nitric oxide synthase (iNOS), Cyclo-oxygenase-2 (COX-2), and aldolase reductase in an AOM-induced colon cancer rodent model [40]. Furthermore, by maintaining glutathione, catalase, SOD, and GSH peroxidase activity through the Nrf2–antioxidant response, PTS showed anti-cancer activity in a UVB-stimulated skin cancer model [41]. In the innate immune system, neutrophils produce reactive oxygen species (ROS) to destroy pathogens with the help of NADPH oxidase, which produces a superoxide anion. The overproduction of ROS can cause tissue damage that is observed in diseases such as rheumatoid arthritis and ischemic injury. PTS lowered the neutrophil count in arthritic animals and facilitated a mild decrease in ROS production, with a limited effect on neutrophil activity [42].

In a dose-dependent manner, PTS has been identified to exhibit a potent antioxidant effect against several free radicals, including 2,2-Diphenyl-1-picryl-hydrazyl (DPPH), 2,2′-Azino-bis 3-ethylbenzothiazoline-6-sulfonic acid (ABTS), hydroxyl, superoxide, and hydrogen peroxide. Furthermore, PTS treatment is associated with increased antioxidant enzymes, such as SOD and GPX, via Nrf2 activation in neuronal cells [43]. In Alzheimer’s disease models, PTS increased PPAR-α, a modulator of neural antioxidant activities [43]. 

### 5.2. Pro- and Anti-Apoptotic Pathways

PTS inhibited cell proliferation and acted as an active inducer of apoptosis in certain cancerous cell lines [8]. Moreover, PTS treatment induced caspase release and O_2_^−^ production, which depolarizes the mitochondrial membrane, triggering the intrinsic mitochondrial-derived apoptosis of cancerous cells [44,45]. Chakraborty et al. identified that PTS modified markers associated with mitochondrial apoptosis and improved the expression of the antioxidant enzymes GPx, GR, and GSH in an in vitro prostate cancer model [46]. Moreover, PTS induced apoptosis in gastric adenocarcinoma cells through the increased upregulation of cytochrome C, Bad, Bax, and caspases [47]. Genomic analysis revealed that PTS treatment in pancreatic cancer upregulated pro-apoptotic genes and anti-proliferative markers [48].

**Figure 2:**
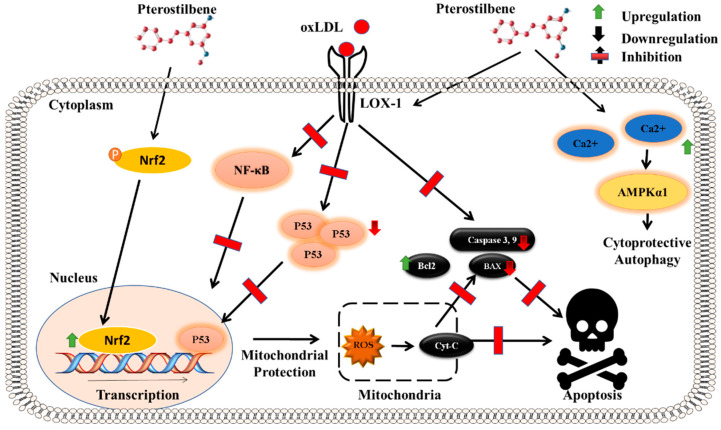
**Anti-apoptotic pathway of pterostilbene:** PTS mediates its cytoprotective effect mainly through the activation of Nrf-2, which in turn protects the mitochondrial functioning, thereby causing a reduction in the induction of pro-apoptotic factors such as cytochrome C, BAX, and caspase-3. In vascular endothelial cells, PTS protects against the initiation of apoptotic signaling by countering the effect of oxLDL in activating its receptor lectin-like oxidized low-density lipoprotein (LOX-1), thereby preventing the accumulation of P53 as well as the activation of NFκB. Moreover, PTS increases the intracellular calcium levels and promotes the cytoprotective autophagy of the cell, consequently preventing the deleterious effect of apoptosis.

Interestingly, PTS was reported to inhibit the effects of apoptosis in vascular endothelial cells [49]. Apoptosis induces plaque instability in atherosclerosis, where oxLDL (oxidized low-density lipoprotein) triggers the apoptosis of VEC by activating lectin-like oxLDL receptor-1. PTS inhibits the apoptosis induced by oxLDL and stimulates cytoprotective autophagic cell death in VECs, thereby dampening the atherosclerotic effect of oxLDL [50]. Notably, this effect was achieved by increasing the accumulation of intracellular calcium, followed by the subsequent activation of the AMPK α1 subunit (AMPKα1) [50]. PTS also suppressed the oxidative damage induced by oxLDL by reducing the mitochondrial membrane potential and lowering the levels of pro-apoptotic proteins such as Bax and p53 [49]. PTS administration to cochlear cells obtained from STZ-induced diabetic rats demonstrated the protection of the cochlea from ototoxicity through the inhibition of apoptosis [51]. Interesting work from our lab deduced that cytoprotection by PTS against cytokine-induced cellular damage in MIN6 mouse pancreatic cells involves the activation of Nrf2 signaling, associated with the inhibition of pro-apoptotic signaling through the attenuation of the BAX/Bcl-2 ratio and the reduced activity of caspase-3 [36]. Our evidence indicates that the PTS-mediated anti-apoptotic effect is also a consequence of Nrf-2 activation (Figure 2).

### 5.3. Anti-Inflammatory Pathway

**Figure 3:**
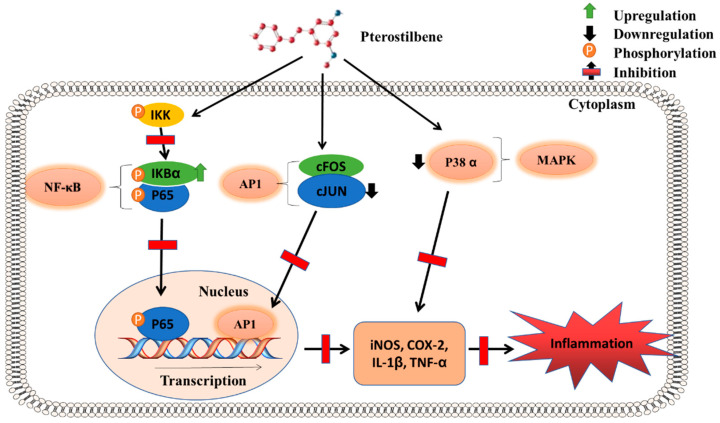
**Anti-inflammatory pathway of pterostilbene**: PTS mediates its anti-inflammatory effect mainly through the inhibition of the transcription factors NFκB and AP-1, which leads to the attenuation of downstream pro-inflammatory mediators, including TNF-α. Further, PTS also inhibits P38 mitogen-activated protein kinase (MAPK) signaling and prevents the induction of iNOS and COX-2.

PTS possesses strong anti-inflammatory properties [27], with iNOS, COXs, leukotrienes, NF-κB, Tumor Necrosis Factor Alpha (TNF-α), and Interleukin-1 beta (IL-1β) reported as its primary targets [52]. Endoplasmic reticulum (ER) stress plays a vital role in inducing endothelial cell inflammation. In human umbilical vein endothelial cells (HUVECs) stimulated by TNF-α, PTS attenuated inflammatory cytokine production and inhibited monocyte adhesion. Importantly, PTS treatment also countered the ER-stress-related molecules stimulated by TNF-α [53]. In HT-29 colon cancer cell lines, PTS mediated the anti-inflammatory pathway through the inhibition of the protein kinase cascade activated by p38 mitogen and led to the suppression of pro-inflammatory cytokine production [54]. In canine chondrocytes, treatment with PTS decreased Matrix Metalloproteinase (MMP)-3, sGAG, and TNF-α, thereby exhibiting anti-inflammatory properties [8]. When combined with cyclodextrin, PTS treatment inhibited biofilm formation by *F. nucleatum* in periodontitis [55]. Importantly, PTS was identified to inhibit the NF-κB-induced inflammatory response by preventing its nuclear translocation. Indeed, PTS treatment in the TPA-induced mouse epidermis led to decreased IkappaB kinase (IKK) activity and improved the retention of IkappaBa (IKB-α), which ultimately blocked the nuclear translocation of NF-κB [56]. Another significant pathway through which PTS inhibited inflammation was through the marked attenuation of the transcription factor activator protein-1 (AP-1) by affecting the binding of the c-JUN subunit to AP-1 response elements [56] (Figure 3).

While studying the anti-inflammatory effects of PTS on ischemia/reperfusion injury in a middle cerebral artery occlusion (MCAO) rodent model, it was found that treatment with PTS suppressed the swelling and disintegration of cells, the infiltration of macrophages and monocytes, and the degranulation of polymorphonuclear leukocytes, thereby exhibiting a neuroprotective effect through an anti-inflammatory mechanism [57]. Moreover, PTS dampened the astrocyte-mediated inflammatory and oxidative damage caused by ischemia/reperfusion injury through the inhibition of NF-κB [58]. The effect of PTS on lipopolysaccharide-induced pulmonary fibrosis was identified to involve the activation of Keap-1/Nrf2, the inhibition of caspase-dependent A20/NF-κB and NLRP3 signaling pathways, and the suppression of inflammation [59]. Furthermore, PTS exhibited a protective role in arthritis induced by Freund’s adjuvant (CFA) in rats by suppressing inflammatory mediators and cytokines [60].

## 6. Therapeutic Properties of PTS

**Figure 4:**
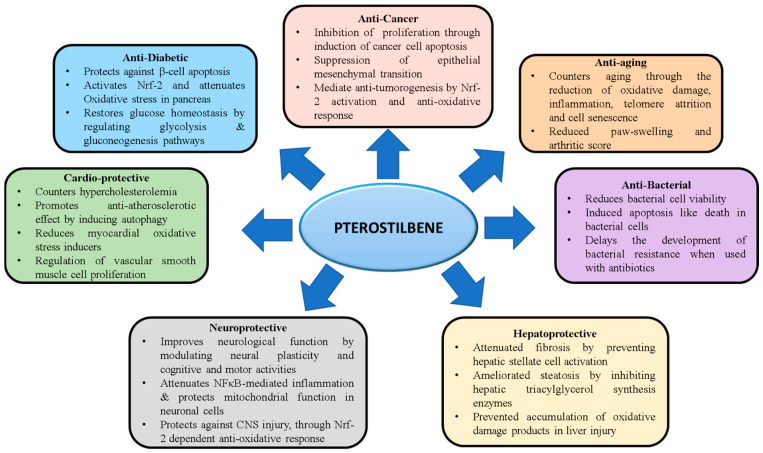
**Therapeutic properties of PTS in various disease conditions**.

Various therapeutic properties of PTS have been documented since its discovery. The phytonutrient is reported to have potent anti-cancer, anti-inflammatory, immunomodulatory, anti-diabetic, antioxidant, analgesic, anti-obesity, neuroprotective, and anti-aging properties [9] (Figure 4).

### 6.1. Anti-Cancer Activity of PTS

Several experimental studies have demonstrated the inhibitory effects of PTS against various cancer cells, including stomach, skin, lung, liver, breast, colon, pancreas, oral, lymph, cervical, endometrial, hematological melanoma, prostate, leukemia, and myeloma tumor cells [29,30] (Table 2). PTS has been identified to be useful in preventing and treating cancer by regulating pro-apoptotic or non-apoptotic anti-cancer activities [33]. The development and progression of cancer involve various factors: a group of drug-metabolizing enzymes, cytochrome P450. These enzymes mediate the metabolic activation of several pro-carcinogens and play a crucial role in the inactivation and activation of anti-cancer drugs. CYP1A1 and CYP1B1 are members of the CYP1 superfamily and have important roles in cancer progression. PTS acts as an efficient inhibitor of CYP1A1, CYP1A2, and CYP1B2 in a competitive manner. The anti-proliferative mechanisms of PTS are seen in different concentrations for different cell types [3]. 

Rimando et al. studied the cancer chemopreventive activity of PTS using a mouse mammary gland culture model and showed that PTS (ED50 = 4.8 µM) markedly reduced DMBA-induced mammary alveolar precancerous lesions through its peroxy-radical scavenging antioxidant activity [62]. In mutant p53-breast cancer cell lines MDA-MB-231 and T-47D, PTS facilitated the reduction in oncogenic β-catenin, mTOR, and mutant p53, as well as increased the expression of the pro-apoptotic Bax protein [87]. Moreover, in MDA-MB-231 xenograft mouse models, PTS suppressed the epithelial-to-mesenchymal transition (EMT) through the upregulation of miR-205 and the consequent reduction in pro-EMT src signaling [63]. Notably, PTS exhibited additive anti-cancer effects in combination with other natural compounds. A combination of α-tocopherol succinate (42 and 99 IU/kg) and PTS (40 μg/kg) attenuated the invasive capability of MDA-MB-231 cells [68]. The treatment of ER-positive breast cancer with a combination of tamoxifen (5 μM) and PTS (10 and 20 μM) also exhibited additive effects. In the studied cell lines (MCF7 and ZR-751), the suppression of cancer cell proliferation along with an elevation in apoptotic activity was observed when this combination was employed [45]. 

In the human colorectal adenocarcinoma cell line HT-29, PTS (≥10 µM) inhibited cell proliferation while inducing G1 cell arrest. Moreover, PTS treatment stimulated apoptosis through the attenuation of the STAT3 and AKT kinase signaling pathways [88]. In a rodent model of azoxymethane (AOM)-induced colon cancer, the intake of PTS (40 ppm) through the diet for 45 weeks led to a reduction in tumorigenesis and diminished the levels of proliferating cell nuclear antigen (PCNA), cyclin D1, and β-catenin [89]. PTS was also observed to mediate the anti-cancer effect through the stimulation of Nrf2 signaling and its target genes (HO-1 and GR), which counter the effect of NF-κB-mediated pro-inflammatory signaling. Studies have shown that the overexpression of iNOS and COX-2 is markedly correlated with the progression of colon cancer. Moreover, in an in vitro study using the HT-29 colon cancer model, PTS inhibited the transcriptional expression of augmented iNOS levels and moderated the inhibition of COX-2 in a concentration-dependent manner [40,89]. 

PTS in combination with quercetin at 20 mg/kg/day inhibited the metastatic activity in B16-F10 melanoma by reducing the adhesion of B16-F10 cells to the endothelium and also downregulated the levels of Bcl-2 in cancerous cells [90]. PTS (10 to 50 μM) suppressed the cancer cell proliferation and initiated apoptotic signaling through the induction of lysosomal membrane permeabilization in A375 melanoma cells [91]. Moreover, through the attenuation of iNOS and COX-2 expression, PTS prevented DMBA- and TPA-induced skin tumor formation [92]. Similarly, in rodent models of UVB-induced skin cancer, the anti-cancer activity of PTS was observed to include the prominent induction of Nrf2-mediated antioxidant signaling, resulting in glutathione level maintenance and the improved activities of catalase, SOD, and GPX [41]. Intravenously administered PTS suppresses human melanoma and pancreatic cancer growth in small animals. Evidence indicates an indirect mechanism of cancer growth inhibition, where PTS inhibits pituitary adrenocorticotropic hormone production, mediates the downregulation of glucocorticoid receptors, and stimulates the Nrf2-dependent cancer antioxidant defense system and the stress-related neuroendocrine signaling mechanism [30,69].

Multiple myeloma (MM) models, xenograft mouse models for hematological cancers, and several diffuse large B-cell lymphoma (DLBCL) models have been utilized to study the anti-cancer effect of PTS [64,65]. In the DLBCL cell line, the viability of the cancer cells was largely dependent on the concentration of PTS and was associated with reduced mitochondrial membrane potential, elevated free-radical generation, and caspase-mediated apoptosis when PTS was intravenously administered [64]. In MM cell lines, a similar concentration-dependent suppression of the proliferation of cancer cells was observed through increased caspase activation, further highlighting the anti-cancer properties of PTS [65]. Additionally, PTS treatment was reported to show benefits against Cholangiocarcinoma (CCA), also known as biliary tract cancer, as evidenced by its cytotoxic effects, mediated through autophagy and the inhibition of CCA tumor growth, in two different CCA cell lines [70].

In endometrial cancer cells, the combination of PTS and megestrol acetate produced a synergistic effect through the inhibition of cell-cycle regulators, including cyclin D1, cyclin B1, and CDK4 [93]. An open-label randomized Phase II clinical trial is underway to study the effect of PTS with megestrol acetate in endometrial cancer patients or patients with endometrial complex atypical hyperplasia who are scheduled for hysterectomy (ClinicalTrials.gov Identifier: NCT03671811) (Table 3). Furthermore, PTS suppresses cell-cycle progression and apoptosis in ovarian cancer cells (OVCAR-8 and Caov-3 cells) through the inhibition of the STAT3 pathway. PTS decreased the expression of cell-cycle and anti-apoptotic proteins involved in the STAT3 pathway, including Mcl-1, Bcl-2, and cyclin D1 [94].

There are scientific reports on the therapeutic potential of PTS in attenuating hepatocellular carcinoma (HCC), which is the second-most prominent cause of cancer-related mortality. In a recent study, PTS treatment was reported to inhibit tumor growth and cell proliferation in a dose-dependent manner in an animal model of HCC [96]. A combination of diethylnitrosamine and carbon tetrachloride was used to induce HCC in mouse livers. PTS treatment was demonstrated to upregulate caspase-3 activity and thereby induce apoptosis in HCC tumor tissue. Interestingly, PTS was identified to reduce HCC proliferation through a reduction in SOD2 and the induction of ROS-mediated mitochondrial apoptotic pathways [96]. Further, it was observed that PTS conferred protection against HCC proliferation and inhibited Hepatitis B virus proliferation in several HCC cell lines. Of note, PTS exhibited antiviral and anti-cancer activity in HCC cells that were resistant to Sorafenib (anti-cancer drug) and Lamivudine, an antiretroviral drug [97]. Importantly, the researchers identified that PTS exhibited anti-cancer and anti-retroviral effects through the potent inhibition of ribonucleotide reductase (RR), which plays a critical role in cellular DNA synthesis. Additionally, PTS treatment was demonstrated to markedly inhibit the growth of an HCC xenograft in nude mice with minimal toxicity [97]. PTS was reported to suppress the invasion and growth of HCC by down-regulating the expression of Metastasis-Associated Protein 1 (MTA1) and histone deacetylase 1 (HDAC1) while upregulating the acetylation of the tumor suppressor protein PTEN [98]. The epigenetic-level regulation by PTS could open new avenues in understanding its anti-cancer activity.

### 6.2. Anti-Diabetic Activity of PTS

Diabetes is a disease characterized by uncontrolled sugar levels due to insufficient secretion and the improper action of insulin, usually disturbing the metabolism of fats, carbohydrates, or proteins [99]. Various rodent models have demonstrated the anti-diabetic effect of PTS (Table 2). The compound has been reported to strongly influence glucose homeostasis by decreasing systemic glucose levels while increasing insulin concentrations [3,100]. Indeed, the findings from our lab indicated that PTS treatment markedly regulated blood glucose by improving insulin secretion in STZ-induced diabetic mice [37]. In particular, we observed that PTS-mediated glucose regulation is achieved by regulating glucose metabolism enzymes in the liver of STZ-induced diabetic mice [37]. The oral administration of PTS to diabetic rats elevated the levels of the hepatic glycolytic enzyme hexokinase, reduced the levels of glycogenic enzymes glucose-6-phosphatase and fructose-1,6-bisphosphatase, and thereby improved the peripheral utilization of glucose [72]. PTS also improved the antioxidant capacity in diabetic rats by upregulating GST, SOD, GPX, and catalase levels and counteracting ROS accumulation [100]. These mechanisms protect renal and hepatic cells from the deleterious effects of hyperglycemia-induced oxidative stress. Hence, PTS exhibits anti-diabetic activity by reducing hyperglycemia, but it also protects liver and kidney cells from hyperglycemia-associated damage [100]. 

While investigating PTS-mediated protection against β-cell apoptosis in STZ-induced diabetic rodents, our lab demonstrated that PTS treatment improved glucose homeostasis while attenuating the pro-inflammatory cytokine response. The cytoprotection of β-cells by PTS treatment was conferred by an Nrf2-mediated mechanism, as evidenced by the attenuation of caspase-3 activity and the BAX/Bcl-2 ratio (Figure 2). We also found the inhibition of iNOS and reduced nitric oxide (NO) synthesis in the diabetic pancreas. PTS significantly alleviated the function of pancreatic β-cell cells and improved their survival in the background of cytokine stress, thereby preventing the pathogenic features of STZ-induced diabetes [36]. Furthermore, our proteomic study demonstrated the molecular mechanisms involved following PTS administration in diabetic rodents by employing electrospray ionization tandem mass spectrometry (LC-MS/MS). Our findings indicated that the administration of PTS normalized the levels of 315 proteins that were modulated in diabetic mice. Outstandingly, a major proportion of these proteins were involved in the regulation of redox imbalance, the antioxidative stress response, the unfolded protein response, and ER degradation pathways, indicating that PTS treatment plays a crucial role in the rehabilitation of defective metabolic processes and stress sensors in diabetes [101].

Lipid peroxidation is a characteristic of diabetes, and lipid peroxidation products can damage DNA and contribute to extra-pancreatic tissue damage in diabetes. PTS significantly reduced lipid peroxidation levels and was reported to scavenge DPPH free radicals and peroxyl radicals (ROO*). In a tertiary-butyl hydroperoxide (TBHP)-induced oxidative damage rodent model, free radicals, including hydroxyl, superoxide, and hydrogen peroxide, were attenuated by PTS in a concentration-dependent manner [102]. Notably, in diabetic-nephropathy-induced rats, PTS ameliorated renal damage by dampening the NF-κB inflammatory signaling pathway and inhibiting oxidative stress [73]. Diabetic retinopathy is associated with pathogenic alterations in the structure of the retina, mediated through high glucose levels. PTS was observed to reduce ROS generation while increasing the SOD levels to scavenge free radicals and thereby suppress diabetic retinopathy [38].

### 6.3. Therapeutic Effect of PTS in Liver Diseases

Liver fibrosis results from the overt and chronic accumulation of extracellular matrix proteins, resulting in the scarring of hepatic tissue and the marked disruption of hepatic vasculature, which can ultimately lead to cirrhosis [103]. Along with an increased risk of mortality, cirrhosis is also a risk factor for developing hepatocellular carcinoma [103]. Studies employing both acute and chronic liver injury models have identified the alleviation of liver injury post-PTS administration (Table 2). Of note, PTS treatment administered to a dimethylnitrosamine (DMN)-induced liver fibrosis model in Sprague-Dawley rats reduced DMN-induced changes and attenuated pro-fibrogenic hepatic stellate cell activation. PTS also exhibited hepatoprotective activity through the inhibition of TGF-b1/Smad signaling [75]. 

NAFLD is a chronic progressive liver disorder in metabolic syndromes such as obesity and insulin resistance caused by excessive fat accumulation (hepatic steatosis). PTS administration in Zucker rats showed reduced insulin resistance and attenuated hepatic triacylglycerol levels, thus reducing liver steatosis. Importantly, treatment with PTS reduced hepatic steatosis from grade 2 to grade 1. Of note, PTS was observed to reduce the triacylglycerol synthesis capacity of the liver through a reduction in fatty acid disposal and through the inhibition of triacylglycerol synthesis enzymes such as DGAT2. The PTS-treated rats had improved fatty acid profiles, attributed to its delipidating effect [76]. Furthermore, PTS and its derivative 3′-Hydroxy-pterostilbene reduced NAFLD pathogenesis induced by free fatty acids and a fat-rich diet through the upregulation of SIRT1/AMPK and insulin signaling pathways and the downregulation of the protein expression of SREBP-1, which results in the activation of the β-oxidation of fatty acids and the consequent reduction in fatty acid synthesis. Moreover, PTS was also observed to promote the growth of vital beneficial microbiota, such as Oscillospira, while down-regulating the population of potentially pathogenic bacteria, such as Allobaculum, Phascolarctobacterium, and Staphylococcus [104]. 

Zhang et al. employed an IUGR-induced liver injury model and demonstrated increased circulating alanine transaminase activity, an elevated hepatocyte apoptosis rate, and ROS generation and accumulation. PTS administration reduced these pathogenic processes by preventing the accumulation of hepatic superoxide anions, 8-hydroxy-2 deoxyguanosine, and 4-hydroxynonenal-modified protein by stimulating the translocation of Nrf2 to the nucleus and inducing the antioxidant enzyme SOD2 [39]. Further, in a study investigating the efficacy of PTS against obesity, it was found that PTS formed three hydrogen bonds with the amino acids of PPAR-α, thereby inducing its expression in the livers of BBPX hamsters and lowering the plasma LDL concentration. Furthermore, PTS was also observed to regulate fatty acid oxidation by dose-dependently elevating the phosphorylation of 5′-AMPK [77].

### 6.4. Effects of PTS on Diseases of the Central Nervous System

The antioxidant and anti-inflammatory properties of PTS have been reported to be therapeutic for the aging brain. Evidence from experimental studies indicates that PTS confers protective benefits against Alzheimer’s disease (AD) and vascular dementia [105] (Table 2). PTS treatment potently modulated cognitive impairment and cellular stress. This effect was closely linked to the presence of methoxy groups, which increases lipophilicity. It also positively modulates cellular stress markers by upregulating PPAR-α expression [82]. 

Joseph et al. administered PTS to rats at various doses and measured its concentration in blood plasma and brain tissue (hippocampus). The amount of PTS in the hippocampus was directly related to the intake of PTS and alleviated cognitive function through the modulation of neural plasticity and motor activity. When administered at high doses, the compound was detected in the serum and brain tissue; however, low doses were only found in the serum and not detected in brain tissue [105]. Dose studies are warranted to further understand the threshold dosage for PTS to cross the BBB. 

The induction of the NF-kB signaling pathway is a vital pathogenic component in neurodegenerative diseases. Through the downregulation of NF-kB, PTS limited the inflammatory response in the CNS [82]. Cerebral ischemia/reperfusion injury is a period of impaired blood supply to the brain during an ischemic stroke. Five days of PTS treatment (10 mg/kg) in a common carotid artery occlusion model markedly elevated the membrane potential of mitochondria and induced cytochrome c expression, as well as complex I and IV activity. PTS attenuated the ROS generated by mitochondria and reduced the cytochrome c levels in the cytosol. Considering that HO-1 signaling exhibits protection in Parkinson’s, Alzheimer’s, and other neurodegenerative diseases, the upregulation of HO-1 expression by PTS exhibited cerebral protective effects [81]. 

PTS confers neuroprotection to neuronal Sh-SY5Y cells by reviving estrogen-receptor-α-induced signaling [106]. A reduction in high-glucose-induced CNS injury and mitochondrial-dysfunction-derived oxidative stress was observed upon PTS administration due to the activation of Nrf2 in hippocampal neuronal cells [107]. However, high doses of PTS or resveratrol inhibited the physiological immune response to pathogens [108,109,110]. Further dose-dependent studies are required to identify the appropriate PTS dose to achieve the therapeutic effect. 

### 6.5. Effects of PTS on Cardiovascular Diseases

Hypercholesterolemia is associated with an increased risk of cardiovascular diseases. PTS treatment was demonstrated to reduce atherosclerosis and myocardial infarction in animal models of cardiovascular diseases (Table 2). PTS treatment lowered plasma lipoproteins and cholesterol, protecting vascular endothelial cells from oxidation and promoting cytoprotective macroautophagy [7]. pTeroPure, a highly purified trans-PTS patented by Chromadex, Irvine, CA, has been proven to significantly reduce blood pressure in adults [2]. The combination of PTS and hydroxypropyl-β-cyclodextrin improved cardiac function in an experimental monocrotaline (MCT)-induced/arterial-hypertension-provoked right-heart-failure model through the induction of the antioxidative response. In particular, PTS enabled the rehabilitation of glutathione metabolism and restored redox homeostasis in the right ventricle of MCT-treated rats. At higher doses, PTS attenuated lipoperoxidation and total phospholamban while increasing the levels of sarcoplasmic reticulum calcium ATPase (SERCA) in the right ventricles of diseased rodents [83].

An elevation of mechanical stress in the endothelium puts the heart at risk of injury to its vasculature and thrombogenesis, which is worsened by oxidative stress. The endogenous antioxidative response of the vascular system is responsible for exerting a protective effect by attenuating oxidative damage; however, the antioxidant capacity may become exhausted due to increased and chronic exposure to ROS, creating an imbalance between oxidant and antioxidant activities. In an ischemia/reperfusion-induced myocardial damage experimental model, PTS showed a cardioprotective effect by reducing myocardial peroxynitrite, superoxide production, malondialdehyde content, and NADPH oxidase enzyme expression and by increasing the antioxidant SOD activity to protect against oxidative stress [111].

The unchecked proliferation of vascular smooth muscle cells leads to atherosclerosis and the consequent development of vascular stenosis [112]. In atherosclerosis, PTS has been reported to exhibit protective effects through the modulation of vascular smooth muscle cells (VSMCs) and VECs through the blocking of an Akt (a serine/threonine kinase)-dependent pathway. In a platelet-derived growth factor (PDGF)-BB-induced VSMC proliferation model, PTS treatment downregulated the promoters of DNA synthesis and VSMC proliferation, including cyclin-dependent kinase (CDK)-2, CDK-4, cyclin E, cyclin D1, retinoblastoma (Rb), and proliferative cell nuclear antigen (PCNA) [113]. 

### 6.6. Effects of PTS on Aging

Polyphenols have been extensively documented to protect against aging and age-related diseases such as atherosclerosis, arthritis, cataracts, osteoporosis, diabetes, and neurodegenerative and cardiovascular disorders. Studies indicate that PTS acts as an anti-aging agent by regulating hallmark features, including oxidative damage, inflammation, telomere attrition, and cell senescence [43] (Table 2). Owing to its ability to cross the BBB, PTS can localize within the brain and provide potential therapeutic benefits against age-related neurodegenerative disorders [108]. Indeed, PTS countered lipopolysaccharide-induced microglial activation in rodents and ameliorated learning and memory impairments [114]. PTS was also demonstrated to effectively reverse aging-associated behavioral deficits in rats. Indeed, the concentration of PTS in the rat hippocampus was directly correlated with dopamine release and working memory [105].

Employing the SAMP8 mouse, which is increasingly being recognized as an effective model of accelerated aging in the background of sporadic and age-related AD, Chang et al. demonstrated that dietary doses of PTS exhibited more potency when compared to resveratrol in modulating cognitive behavior and cellular stress [82]. Notably, the study attributed the anti-aging effect to the activation of PPAR-α. Moreover, PTS was also reported to extend the lifespan of SAMP8 mice, an effect attributed to c-Jun N-terminal protein kinase inhibition [115].

Ocular surface inflammation is a multifactorial disease that is particularly prevalent among the elderly. PTS has been reported to restore the imbalance between oxygenases and antioxidative enzymes through the attenuation of COX-2 and the upregulation of SOD1 and peroxiredoxin-4 (PRDX4) activities in the background of hyperosmotic stress [116]. PTS treatment is associated with a reduction in oxidative damage mediators, including malondialdehyde (MDA), 4-hydroxynonenal (4-HNE), aconitase-2, and 8-hydroxydeoxyguanosine (8-OHdG) levels, in a human corneal epithelial cell model induced by hyperosmotic medium stress [116]. Blueberry consumption has been reported to prolong the lifespan and improve thermo-tolerance in C. elegans [117]. In D. melanogaster, blueberry extracts upregulated the expression of the antioxidant enzymes SOD and catalase, which were mainly attributed to lifespan extension [118]. Dietary supplementation with blueberries, which contain polyphenols such as PTS, alleviated the damaging effect of aging on motor behavior and neuronal signaling and lowered the amyloid-beta content in a transgenic AD rodent model [119]. In an open-label, single-arm, monocentric study investigating the efficacy of Pon skin brightening and PTS anti-aging, a cream formulation containing 0.4% PTS was highly effective in reducing aging markers and brightening the skin tone of study participants. Furthermore, employing an in vitro experiment, PTS was reported to exhibit anti-tyrosinase activity and inhibit melanogenesis, which could have contributed to the reduction in the markers of skin aging [120].

Arthritis is characterized by the painful swelling of joints, which worsens with age. In a Freund’s adjuvant (CFA)-induced arthritis model in rats, PTS significantly reduced paw swelling, the arthritic score, and body weight. Interestingly, it also helped restore the healthy gut microbiota ecosystem by reducing the relative abundance of Helicobacter, Desulfovibrio, Lachnospiraceae, and Mucispirillum. Considering the evidence of PTS in suppressing inflammation through intestinal bacteria alterations, studies investigating its therapeutic potential against inflammatory bowel disorders could be of clinical value [60].

### 6.7. Antibacterial Effect of PTS

Stilbene compounds, including PTS, are natural antibacterial agents due to their low hydrophilicity, enabling them to penetrate hydrophobic biological membranes [121]. PTS with cyclodextrin exerts antimicrobial effects by inducing bacterial cell content leaks, resulting in a reduction in bacterial cell viability. It also inhibits *F. nucleatum* biofilm formation, making it a potential candidate for treating periodontitis [55]. Bacillus cereus, a foodborne pathogen contaminating uncooked food, was tested with PTS. Following treatment, apoptosis-like cell death (ALD) was induced and increased intracellular ROS in bacterial cells. Additionally, an improvement in the beneficial gut microbiota Bacteroidetes was also documented [122]. 

PTS, along with gentamicin, was tested against six Gram-positive and Gram-negative bacteria, and the combination was found to be synergistic against three susceptible strains, *Staphylococcus aureus* ATCC 25923, *Escherichia coli* O157, and *Pseudomonas aeruginosa* 15442. However, no significant difference was observed from gentamicin treatment alone. Bacterial growth was fully diminished after 2–8 h treatment with PTS and gentamicin, exhibiting the potential to delay the development of bacterial resistance by utilizing lower concentrations of antibacterial agents [123]. Methicillin-resistant S. aureus (MRSA) is a multi-drug-resistant S. aureus strain, whose biofilm thickness was reduced from 18 to 10 μm when treated with PTS. Topical administration ameliorated the abscess formation induced by MRSA, thereby lowering the bacterial burden and improving the architecture of the skin [124]. PTS has also been used to treat infections with Staphylococcus spp. or Enterococcus faecalis in biofilms due to a reduction in the growth capacity of Gram-positive cocci [121]. 

### 6.8. Therapeutic Effects of PTS against COVID-19 Infection

The COVID-19 pandemic, caused by severe acute respiratory syndrome coronavirus 2 (SARS-CoV2), triggered a major setback to global human health and the economy in the 21st century [125]. Resveratrol was tested and proved to have effective therapeutic value against MERS-CoV infection by decreasing cell death [126]. Screening studies have indicated that stilbenes inhibit complex formation between the spike protein and ACE-2 receptor, thereby blocking viral entry into the host cell [127]. Based on these studies, stilbene derivatives could be considered important drug candidates for COVID-19 [127]. PTS has been demonstrated to actively inhibit SARS-CoV-2 virus replication in infected African green monkey kidney cells. Antiviral activity was seen for up to five rounds of replication, which indicates a long-lasting therapeutic effect.

Moreover, in human primary bronchial epithelial cells isolated from healthy volunteers, PTS showed an antiviral effect for up to 48 h after infection. These data promote the use of PTS as a potent drug against COVID-19 and warrant further clinical trials to prove its antiviral efficacy early in COVID-19 [125]. Furthermore, PTS, co-administered with zinc, has also been identified as a potential COVID-19 adjuvant therapy for managing moderate–severe disease [128]. However, further clinical trials are required to back the pharmacotherapeutic potential of PTS. COVID-19 patients with comorbidities related to metabolic syndromes, such as diabetes, obesity, hypertension, and cardiovascular disease, have low levels of the HO antioxidant enzyme. Higher HO-1 expression has been associated with reduced susceptibility to COVID-19 infection [129]. Considering that COVID-19 patients are susceptible to the induction of overt inflammatory processes and cytokine storms, PTS treatment could exert anti-inflammatory and cytoprotective effects by increasing HO-1 expression [130]. 

## 7. Enhancement of PTS Bioavailability

The phenolic group at the 4′ position of PTS is an attractive target for phase 2 conjugative enzymes, which negatively impacts its bioavailability. PTS is also insoluble in water, limiting its concentration in the aqueous environment [77]. Safe, bioavailable formulations of PTS can help increase its bioavailability in the body and reduce known and unknown side effects [30,131]. Some of the ongoing trials for PTS include using dietary blueberry supplements, 250 mg of Nicotinamide Riboside, and 50 mg of PTS, commercially known as Basis™, developed by Elysium, or ElevATP, a combination of polyphenols developed by FutureCeuticals (Table 3). Further clinical trials are warranted to improve the therapeutic bioavailability and optimal administration methods of PTS.

Crystal engineering has been a promising field for bioavailability enhancement due to the advantageous, unique solubility and dissolution rates of drugs [132]. Formulations of cocrystals can improve the dissolution profile and bioavailability of PTS with improved performance. Notably, picolinic acid, an endogenous L-tryptophan metabolite, exhibits neuroprotective, immunological, and anti-proliferative properties. The oral administration of PTS–picolinic acid cocrystals to rats revealed a 10-fold amplification of the bioavailability of PTS when compared to solid oral forms [131]. PTS–caffeine cocrystal solubility was also observed to have over 27 times higher solubility [132]. 

In the form of a prodrug, a bis (hydroxymethyl) propionate-based analog of PTS exhibited superior tumor inhibitory activity in cisplatin-resistant oral squamous (CAR) cancer cell lines. An increase in absorption, a reduction in metabolism, and the maintenance of high concentrations of PTS were some of the observed benefits of prodrug administration. The concentration of PTS in the blood was significantly higher when the amino acids isoleucine or β-alanine were utilized. Oral gavage administration of PTS with megestrol acetate at 10 mg/kg/day reduced the tumor weight and volume in endometrial cancer rats [61]. 

Notably, the chronic administration of PTS facilitated burn-wound healing in diabetes, facilitated by a significant reduction in diabetes-induced oxidative stress and the suppression of hypoxia-induced factor1α (HIF1α) activity [130]. Liposome-engulfed PTS is also efficient for the topical administration of the drug [41]. Poly (3-acrylamidophenyl boric acid-b-PTS) [p(AAPBA-b-PTE)] is a round nanoparticle of PTS, with a size ranging from 150 to 250 nm. Notably, they possess an appropriate pH and sensitivity to glucose. In vitro and in vivo studies showed that PTS nanoparticles were nontoxic and safe. Upon administration, these nanoparticles were observed to reduce glucose levels, reverse micro-inflammation, and improve the antioxidant profile in mice [133]. Further, antibody-4arm-polyethylene glycol-PT conjugate, as an antibody–drug conjugate, was used for the targeting and co-delivery of drugs to tumors [134]. A composition with Zein/fucoidan composite nanoparticles as carriers of PTS was prepared using an anti-solvent precipitation method. The findings indicated that this method provided a better-controlled release and could be utilized as a potential carrier for the protective encapsulation of PTS [135]. Poly(2-oxazoline)–PTS block-copolymer nanoparticles are also being used for dual anti-cancer drug deliveries in chemotherapy [136]. 

Solubilizing PTS in 2-hydroxypropyl-b-cyclodextrin (HP-b-CD) improved its bioavailability by 3.7 times [25]. Furthermore, a lipid-based encapsulation system has been used to enhance the stability of PTS in the aqueous phase. In particular, encapsulation in nano-emulsions made of flaxseed oil and olive oil was studied by Sun et al. The bio-accessibility was high in the flaxseed-oil nano-emulsion. However, greater amounts of intact PTS were transported across intestinal enterocytes in olive-oil nano-emulsions [137]. The oral administration of zinc pectinate beads, prepared through the ionic gelation method, successfully distributed PTS to the colonic tissue [138]. Interestingly, physical exercise promoted the biosynthesis of the anti-inflammatory mediator MaR1, leading to a preventive effect in rheumatoid arthritis. The oral administration of PTS, when accompanied by moderate physical activity, attenuated the pathological process of rheumatoid arthritis in a bovine type II collagen (BIIC)-stimulated rat model [86].

## 8. Conclusions and Future Prospects

Numerous studies that have evaluated PTS for its therapeutic potential have demonstrated its role as a promising candidate drug for health benefits in a broad spectrum of disease conditions. Various experimental studies have confirmed that PTS has anti-cancer, anti-diabetic, anti-hypertensive, antimicrobial, anti-aging, anti-atherosclerotic, and neuroprotective properties. PTS has been documented to exert its beneficial effects mainly by modulating antioxidant, anti-apoptotic, and anti-inflammatory pathways. Of particular interest, the activation of the Nrf2 signaling pathway by PTS has been an essential focus of our lab. Our findings indicate its potential as a promising drug candidate for diabetes and associated complications. Even at higher doses, PTS did not exhibit toxicity in animal studies, providing further encouragement to explore the use of the compound in more human clinical trials. However, considering that some of the studies have employed the co-administration of PTS in combination with other compounds to improve its therapeutic efficiency, the potential effect of drug interactions should be considered. 

Although PTS has been identified to exert marked therapeutic benefits, most findings have been proven only in experimental models. Human clinical trials have been largely limited due to the less-than-desired bioavailability of the compound. To overcome this limitation, various strategies have been implemented, which involve modifying the administration routes and formulations of PTS, including cocrystals, prodrugs, nanoparticles, lipid-based encapsulation, and beads. The potential effects of many drug interactions with PTS remain unclear. Of note, the mode of administration of PTS seems to play an important role in its bioavailability, as the administration of intravenous doses shows a higher distribution when compared to oral intake. Further research that carefully considers the dose, drug interactions, administration route, disease-specific formulations, and short- and long-term biomedical implications is warranted before the clinical adoption of this promising natural compound.

## Figures and Tables

**Table 1:** Potential Sources of Pterostilbene.

Source	Concentration Range	Reference
Blueberries	9.9–15.1 mg/kg of fresh weight	[13]
Blueberries	15 µg/100 g of weight	[11]
Vaccinium berries	99–520 ng/g of dry sample in *Vaccinium ashei* and *V. stamineum*	[11]
Fungal infected grapes	0.2–4.7 mg/g of fresh weight	[14]
Rabbit-eye blueberry	99–151 ng/g of dry sample	[11]
Deerberries	520 ng/g of dry sample	[11]
Peanut	NA	[12]

**Table 2:** Effect of Pterostilbene in Experimental Models of Various Disease Conditions.

Disease Condition	Experimental Model	Effect of Pterostilbene	Reference
Cancer	Endometrial cancer xenograft model	Reduced weight and volume of tumor	[61]
	DMBA-induced mammary alveolar precancerous lesions in mice	Reduced lesions	[62]
	MDA-MB-231 (breast cancer) xenograft model	Suppressed tumor growth	[63]
	UVB-induced skin cancer in mice	Nrf2-dependent antioxidant response	[41]
	Hematological cancer xenograft model	(a) Increased caspase activation (b) Reduced cell proliferation	[64,65]
	Azoxymethane-induced colon cancer model	Reduced iNOS levels and attenuated crypt formation	[66]
	MIA PaCa-2 xenograft model	Inhibited tumor growth and prominent central necrosis	[48]
	HPV-E6-positive cervical cancer mouse model	(a) Increased apoptosis (b) Downregulated E6 and VEGF expression	[67]
	Breast cancer xenograft mouse model	When coupled with Vitamin E: (a) Inhibited Akt (b) Downregulated cell-cycle proteins	[68]
	Melanoma xenograft mouse model	ACTH downregulation led to decreased Nrf2-mediated defenses	[69]
	Large B-cell lymphoma xenograft mouse model	(a) Reduced mitochondrial membrane potential (b) Increased apoptosis	[64]
	Biliary cancer xenograft mouse model	(a) Inhibited proliferation (b) Induced autophagy	[70]
	Multiple myeloma mouse xenograft	(a) Inhibited cell progression (b) Increased ROS production for apoptosis (c) Improved extracellular-signal- regulated kinases 1/2 and c-Jun N-terminal kinase signaling	[65]
	Xenograft of glioma stem cells in mice	Attenuated GRP78, suppressing tumorogenesis	[71]
Diabetes	STZ-induced diabetic rats	Protected rats from ototoxicity through the inhibition of apoptosis	[51]
	STZ- and Nicotinamide-induced diabetic rats	(a) Increased hepatic glycolytic enzyme hexokinase (b) Reduced the levels of glycogenic enzymes and enhanced peripheral utilization of glucose	[72]
	STZ-induced diabetic rats	(a) Regulated NF-κB signal pathway and inhibited oxidative stress and inflammation (b) Improved renal damage	[73]
	STZ-induced diabetic mouse	(a) Normalized plasma VLDL, LDL, and HDL (b) Reduced lipid peroxidation	[36]
	Diabetic rats	(a) Enhanced the peripheral utilization of glucose (b) Elevated the levels of hepatic hexokinase and hepatic phosphofructokinase	[74]
Liver injury	Dimethyl nitrosamine-induced rats with liver fibrosis	(a) Hepatoprotective activities (b) Inhibited TGF-b1/Smad signaling	[75]
	Zucker rats with liver steatosis	(a) Reduced insulin and hepatic triacylglycerol levels (b) Improved fatty acid profile	[76]
	Hamsters with a High-fat diet supplemented with 8% blueberry by-product	(a) Low plasma LDL (b) Increased phosphorylation of adenosine monophosphate-activated protein kinase	[77]
	Hypercholesterolemic hamsters	Cytoprotective macroautophagy	[7]
	CCl_4_-stimulated hepatic fibrosis rat models	(a) Reduced levels of α-smooth muscle cell actin, desmins, MMP2, and MMP9 (b) Downregulated pro-fibrogenesis through the suppression of TGF-β1 in liver tissue	[78]
	Acetaminophen-exposed rats	Suppressed Acetaminophen-induced oxidative stress	[79]
Diseases of the Central Nervous System	Amyloid precursor protein (APP)/Presenilin 1 (PS1) SERCA mouse model with Alzheimer’s disease	Reduced amyloid-beta content Improved pathological changes	[80]
	Common carotid artery occlusion mouse model to study cerebral ischemia/reperfusion injury	Upregulated antioxidant activity through HO-1	[81]
	SAMP8-Alzheimer’s disease mouse model	Increased peroxisome proliferator-activated receptor-α expression	[82]
	Middle cerebral artery occlusion rodent model	(a) Suppressed the swelling and disintegration of cells and attenuated the infiltration of macrophages and monocytes (b) Attenuated the degranulation of polymorphonuclear leukocytes in neural tissue	[57]
Cardiovascular disease	Monocrotaline-treated rats with reduced cardiac function	(a) Prevented reduction in stroke volume and cardiac output (b) Reduced lipid peroxidation and total phospholamban (c) Increased SERCA expression in the right ventricle	[83]
	Apo-lipoprotein-E-deficient mice	Reduced atherosclerosis by inhibiting lipid peroxidation and enhanced antioxidants	[84]
	Fischer-344 rat model of coronary artery ligation	Reduced myocardial infarction size by 22%	[85]
Arthritis	Bovine type II collagen-stimulated rat arthritis model	Reduced pathological process of arthritis when coupled with physical exercise	[86]
	Freund’s adjuvant (CFA)-induced arthritis rat model	(a) Alleviated the swelling of paw and reduced arthritic score (b) Improved body weight	[60]
	Injection of heat-killed *Mycobacterium butyricum* in Lewis rats	Lowered number of neutrophils, thereby downregulating neutrophil oxidative burst	[42]

**Table 3:** Clinical Trials Involving Pterostilbene.

Clinical Trial	Subjects	The Drug Used/Diet	Status	Findings	Reference
Studying the Effects of ElevATP on Body Composition and Athletic Performance of Healthy Individuals	Healthy 18–35-year-old males	ElevATP with and without caffeine and Vitamins	Completed	Improved strength and power output in the lower body	ClinicalTrials.gov Identifier: NCT02819219
Study of Pharmacokinetics and Safety of Basis™ in Subjects with Acute Kidney Injury	Patients with Acute Kidney Injury	Basis	Completed	Nicotinamide riboside with pterostilbene increased whole-blood NAD + levels	ClinicalTrials.gov Identifier: NCT03176628
Effect of PTS on Cholesterol and Hypertension	Patients with hyperlipidemia and increased Blood Pressure	1. PTS (low dosage of 50 mg and high dosage of 125 mg) 2. Low dose combination of PTS and grape extract (50 mg + 100 mg)	Completed	PTS increased LDL and reduced blood pressure in adults [95]	ClinicalTrials.gov Identifier: NCT01267227
Evaluating Safety and Benefits of Basis™ Among Elderly Subjects	60–80-year-old healthy subjects	Basis™	Completed	Yet to be published	ClinicalTrials.gov Identifier: NCT02678611
A Trial of Nicotinamide/PTS Supplement in Amyotrophic Lateral Sclerosis	Patients with Amyotrophic Lateral Sclerosis	EH301 (nicotinamide riboside/PTS)	Recruiting	Yet to be published	ClinicalTrials.gov Identifier: NCT05095571
Effect of Blueberries in Postmenopausal Women with Elevated Blood Pressure for Improving Vascular Endothelial Function	Postmenopausal women with elevated blood pressure, hypertension, and endothelial dysfunction	Blueberry powder	Active Phase 2 Phase 3	Yet to be published	ClinicalTrials.gov Identifier: NCT03370991
Supplementing Wild Blueberries to Study Cardiovascular Health in Middle-aged/Older Men	45- to 70-year-old men with hypertension and endothelial dysfunction	Blueberry powder	Recruiting Phase 1 Phase 2	Yet to be published	ClinicalTrials.gov Identifier: NCT04530916
Effect of High-intensity Training and Daily Consumption of Basis™ on Muscle Metabolism and Exercise	18- to 25-year-old healthy men	NRPT (125 mg nicotinamide riboside and 25 mg PTS)	Phase 1	Yet to be published	ClinicalTrials.gov Identifier: NCT04050358
Study of Treating Megestrol Acetate with or without PTS in Patients with Endometrial Cancer Undergoing Hysterectomy	Women with Atypical Endometrial Hyperplasia and Endometrial Carcinoma	Megestrol Acetate with or without PTS	Recruiting Phase 2	Yet to be published	ClinicalTrials.gov Identifier: NCT03671811
Protection of Basis™ in Acute Kidney Injury	Patients with Acute Kidney Injury	Nicotinamide riboside + PTS	Recruiting Phase 2	Yet to be published	ClinicalTrials.gov Identifier: NCT04342975
Studying the Benefit of Supplementation with Short-term Curcumin and Multi-polyphenol	Non-smokers in good health not taking medications or dietary supplements	Polyresveratrol supplementation (100 mg of trans-PTS, 100 mg of curcumin phytosome, 100 mg of quercetin phytosome, 100 mg of green tea phytosome, and 100 mg of trans-resveratrol)	Unknown	Not Published	ClinicalTrials.gov Identifier: NCT02998918
Studying effects of Nicotinamide Riboside and PTS on Muscle Regeneration in Elderly Humans	55- to 80-year- old individuals	Nicotinamide riboside/PTS-NRPT (500 mg/100 mg twice daily)	Completed	Not published	ClinicalTrials.gov Identifier: NCT03754842
Evaluating Sirtuin Supplements to Benefit Elderly Trauma Patients to study recovery of function	Individuals 65 years and older presenting to trauma bay	Nicotinamide riboside and PTS	Unknown	Not published	ClinicalTrials.gov Identifier: NCT03635411
Effects of a Seven-day BASIS™ Supplementation on Menopausal Syndrome, Estradiol levels and Measurements of the Urinary Vitamin B3	Women 35 years or older	BASIS™	Completed	Not published	ClinicalTrials.gov Identifier: NCT04841499

## Data Availability

Not applicable.
